# Editorial: Paediatric vestibular disorders – a focussed diagnostic approach for best management outcomes

**DOI:** 10.3389/fneur.2024.1532327

**Published:** 2025-01-03

**Authors:** Soumit Dasgupta, Jacob Brodsky, Josine Widdershoven

**Affiliations:** ^1^Department of Paediatric Audiovestibular Medicine, Alder Hey Childrens National Health Service Foundation Trust, Liverpool, United Kingdom; ^2^Faculty of Health and Life Sciences, University of Liverpool, Liverpool, United Kingdom; ^3^Department of Otolaryngology and Communication Enhancement, Children's Hospital Boston, Boston, MA, United States; ^4^Harvard Medical School, Boston, MA, United States; ^5^Department of Otorhinolaryngology, Maastricht University Medical Centre, Maastricht, Netherlands; ^6^Faculty of Health, Medicine and Life Science, Maastricht University, Maastricht, Netherlands

**Keywords:** pediatric vestibular, vestibular testing in children, balance, dizziness, vertigo

## Introduction

Many conditions can cause vestibular symptomatology in children across several organ systems beyond the ear and often differ from adults. Assessing children for such disorders benefits from a holistic multidisciplinary approach. Accurate diagnosis is essential for favorable treatment outcomes. This Research Topic includes papers from many experts focusing on processes that can guide clinicians toward reliable diagnosis and management of these conditions in children.

## Reviews

Wood et al. described the uncommon entity of vertiginous epilepsy. The authors emphasized that crucial distinctions from other more common vestibular conditions in children, such as vestibular migraine and recurrent vertigo of childhood, may be subtle or difficult to elicit in very young patients. Affected children may present with vertigo lasting from seconds to minutes, often with headache, nausea, vomiting, pallor, staring and/or loss of consciousness. Staring spells were found to be helpful in differentiating vestibular epilepsy from vestibular migraine and other causes of episodic vertigo, as were auditory hallucinations and a family history of seizures. Electroencephalography is a key component of diagnosing this condition, along with other tests where indicated. Anti-seizure medications are typically effective.

Genovese et al. performed a systematic review following PRISMA guidelines observing that vestibular assessment is often overlooked in sensorineural hearing loss (SNHL) algorithms, which may have important implications for motor and cognitive development. Fifty-nine out of 1,209 papers met the inclusion criteria of SNHL with documented vestibular function assessment. Nearly 70% of children with SNHL showed vestibular impairments with severe bilateral deficits in 20–40%. Selection of vestibular tests and listed etiologies of cochleovestibular loss varied greatly. The authors concluded that consensus on an optimal battery of vestibular tests in children with SNHL is needed.

## Cohort studies

Gerdsen et al. studied 86 children with SNHL who underwent multiple vestibular tests using their own normative data. Vestibular hypofunction was identified in 44% of the patients, 60% of whom had bilateral impairments. Caloric testing was found to be the most sensitive and specific of the tests performed with improved sensitivity and specificity when combined with video head impulse testing (VHIT). They recommended a multimodal test battery employing several tests to assess vestibular function in children for optimal diagnostic accuracy.

Dasgupta et al. investigated the suppression head impulse test (SHIMP) in children, which is a new test employing a head thrust with a moving target in contrast to a stationary target with VHIT that requires a voluntary effort to suppress the vestibulo-ocular reflex (VOR). Emerging evidence in adults suggests that SHIMP is a valuable tool to assess vestibular compensation by the morphology of compensatory saccades that are opposite in direction to the VOR weakness saccade. The test may also be a better indicator of VOR gain as it is not contaminated by covert saccades. The authors studied SHIMP in 44 children with vs. without vestibular problems. They observed statistically significant differences in the combined right and left VOR gain, peak saccadic velocities and asymmetry of VOR gain between the two groups. The SHIMP VOR gain was spread across a wide range of values suggesting that vestibular compensation is a dynamic process that influences VOR gain in different stages of vestibular compensation in children. The authors concluded that the SHIMP test was feasible in children yielding meaningful inferences about vestibular compensation and VOR suppression for a diagnosis to influence future rehabilitation.

Ölçek et al. investigated the functional aspect of the vestibular system in 15 children with and without specific learning disability (SLD), respectively, using a pediatric subscale and the functional head impulse test (fHIT), which is slowly gaining popularity with minimal data in the pediatric population. This subjective test measures visual fixation and reading ability during head movement depending on subject response of correctly identifying an optotype with a head impulse in either direction. There was no statistically significant difference between static visual acuities as measured with fHIT or in PBS scores between groups. In terms of correctly identifying optotypes, there was a statistically significant difference between 40,000/s2 on both sides and when all accelerations were combined on both sides. The authors inferred that these differences lend weight to the previously published observation that gaze stabilization may be affected in SLD. They also pointed out that the vestibular system participates in cognitive development in children and thus a functional and objective assessment of the vestibular system is useful in SLD.

Zaubitzer et al. investigated vestibular function in 23 children with documented neonatal sepsis who all underwent gentamycin therapy for at least 5 days using lateral canal vHIT, the Denver Developmental Scale, and a questionnaire for pre-existing or concomitant vestibular disorders and developmental milestones. About a third showed developmental delays, potential balance disorders, or risk factors for a vestibular disease in their medical history, out of which about a third exhibited vestibular weaknesses. The children with abnormalities in vHIT did not show any other clinical signs of balance dysfunction. vHIT was mildly abnormal in 47.8% with unilateral abnormality observed more frequently than bilateral. The authors explained the predominantly unilateral weakness by fatigue generated artifacts or unilateral recovery. They proposed that whilst VOR gains were normal or mildly reduced in their cohort, refixation saccades may indicate vestibular weakness. Normal VOR gain with saccades has been established as a biomarker of compensated vestibular weakness that is more common in children than in adults due to possible vestibular regeneration. They concluded that vHIT is sensitive for detecting high frequency vestibular weakness in children undergoing gentamycin therapy.

## Conclusion

A central theme of the papers in this Research Topic is that effective diagnosis and management of children with vestibular dysfunction depends on a careful clinical history, examination, and diagnostic testing. The absence of such an approach may miss rare entities like vestibular epilepsy and may not recognize the role of subtle vestibular impairments in children with SNHL, motor delays, gentamicin toxicity and even learning disabilities. The recent development of newer, more child friendly tests, such as VHIT, SHIMP, and fHIT, warrants a more uniform consensus as to which series of tests is most appropriate in evaluating the pediatric population.

